# From laparoscopic to robotic-assisted Heller myotomy for achalasia in a single high-volume visceral surgery center: postoperative outcomes and quality of life

**DOI:** 10.1186/s12893-022-01818-2

**Published:** 2022-11-11

**Authors:** Jörn-Markus Gass, Lucien Cron, Francesco Mongelli, Justyna Tartanus, Fiorenzo Valente Angehrn, Kerstin Neuschütz, Markus von Flüe, Lana Fourie, Daniel Steinemann, Martin Bolli

**Affiliations:** 1 Department of General Surgery, Cantonal Hospital of Lucerne, Spitalstrasse, 6000 Lucerne 16, Switzerland; 2 Department of Health Sciences and Medicine, University of Lucerne, 6002 Lucerne, Switzerland; 3 Department of Visceral Surgery, Clarunis, University Centre for Gastrointestinal and Liver Diseases, St. Clara Hospital and University Hospital Basel, Kleinriehenstrasse 30, 4058 Basel, Switzerland; 4 Department of Surgery, Regional Hospital of Lugano, Via Tesserete 46, 6900 Lugano, Switzerland

**Keywords:** Laparoscopic surgery, Robotic-assisted surgery, Heller myotomy

## Abstract

**Background:**

Laparoscopic (LSC) Heller myotomy (HM) is considered the standard procedure for the treatment of achalasia. Robotic platforms, established over the last years, provide important advantages to surgeons, such as binocular 3-dimensional vision and improvement of fine motor control. However, whether perioperative outcomes and long-term results of robotic-assisted laparoscopic (RAL) HM are similar or even superior to LSC technique, especially concerning long-term follow-up, is still debated. Therefore, the aim of the present study was to evaluate intra- and postoperative results as well as long-term quality of life after RAL compared to LSC surgery for achalasia in a single high-volume visceral surgery center.

**Methods:**

Between August 2007 and April 2020, 43 patients undergoing minimally invasive HM for achalasia in a single high-volume Swiss visceral surgery center, were included in the present study. Intra- and postoperative outcome parameters were collected and evaluated, and a long-term follow-up was performed using the gastroesophageal-reflux disease health-related quality of life (GERD-Hr-QuoL) questionnaire.

**Results:**

A total of 11 patients undergoing RAL and 32 undergoing LSC HM were analyzed. Baseline demographics and clinical characteristics were similar. A trend (p = 0.052) towards a higher number of patients with ASA III score treated with RAL was detectable. Operation time was marginally, but significantly, shorter in LSC (140 min, IQR: 136–150) than in RAL (150 min, IQR: 150–187, p = 0.047). Postoperative complications graded Clavien-Dindo ≥ 3 were only observed in one patient in each group. Length of hospital stay was similar in both groups (LSC: 11 days, IQR: 10–13 vs. RAL: 11 days, IQR: 10–14, p = 0.712). Long-term follow-up (LSC: median 89 months, vs. RAL: median 28 months, p = 0.001) showed comparable results and patients from both groups expressed similar levels of satisfaction (p = 0.181).

**Conclusions:**

LSC and RAL HM show similar peri- and postoperative results and a high quality of life, even in long-term (> 24 months) follow-up. Prospective, randomized, controlled multicenter trials are needed to overcome difficulties associated to small sample sizes in a rare condition and to confirm the equality or demonstrate the superiority of robotic-assisted procedures for achalasia. Meanwhile, the choice of the treatment technique could be left to the operating surgeon’s preferences.

## Background

Achalasia is a rare esophageal motility disorder of unclear origin characterized by dysphagia, regurgitation and heartburn [1, 2]. A variety of treatments have been proposed. Oral administration of calcium-channel blockers or nitrates, and local injection of botulinum toxin A provide short term relief. Moreover, balloon dilation is also used, but with modest long-term results [3].

Section of muscle fibers of the esophageal sphincter, the so-called Heller myotomy (HM) currently represents the preferred surgical approach to achalasia treatment. In particular, laparoscopic HM is nowadays widely accepted as safe and effective [4]. However, recurrence rates up to 10–25% and intraoperative esophageal mucosa perforation rates ranging between 4 and 20% are reported in literature [5–8].

Over the last years new techniques have emerged in the surgical endoscopy area with per-oral endoscopic myotomy (POEM) and robotic platforms. Recent reports suggest similar effectiveness of POEM compared to conventional laparoscopic HM, albeit with higher gastroesophageal reflux rates [9, 10]. However, data on long-term outcomes is still missing.

With the introduction of robotic-assisted technology, minimally invasive techniques have become more precise, accurate and safe, particularly for procedures in narrow and confined spaces, thanks to improvements in comfort and maneuverability, high-definition 3-dimensional binocular vision with the option of magnification, and a stable platform with a surgeon operated camera [11]. Therefore, it could reasonably be assumed, that with the robotic systems’ attributed improvement in precision and accurateness, the rate of completely dissected muscle tissue without damage to the esophageal mucosa should be higher. Indeed, both conditions—incompletely dissected muscle layer as well as esophageal perforation—are surmised to result in scar tissue and recurrence of achalasia symptoms.

However, especially in well-established laparoscopic procedures in upper gastrointestinal surgery like fundoplication, bariatric surgery or HM, the lack of superior evidence and the higher costs of the new technique have limited the adoption of robotic-assisted systems [12, 13].

Retrospective studies and subsequent meta-analyses have generally shown at least non-inferiority of robotic-assisted HM compared to laparoscopic techniques concerning intra- and postoperative complications and outcome parameters [14, 15]. However, operation procedures are far from standardized and different technical approaches are utilized even in a single center. Moreover, treatments prior to surgery, body mass index (BMI), American Society of Anesthesiologists (ASA) classification, type of fundoplication and recurrence rates are frequently unreported.

Most worryingly, a majority of these studies have also failed to assess long-term postoperative quality of life representing a main outcome parameter after HM [16, 17].

To address these issues, in this study, including a follow-up of over 2 years, we analyzed postoperative quality of life as well as intra- and postoperative outcomes in a highly homogeneous group of patients undergoing standardized LSC or RAL HM in a single high-volume visceral surgery center.

## Methods

### Inclusion and exclusion criteria

All patients aged ≥ 18 years requiring HM for achalasia with a minimally invasive approach at a high-volume visceral surgery unit in northwestern Switzerland between August 2007 and April 2020 were included in the study. Preoperatively, a gastroscopy, an upper gastrointestinal gastrografin swallow, and, in most cases, an esophageal manometry, were performed to verify and secure diagnosis, and to rule out other reasons for dysphagia.

Between August 2007 and December 2015 all procedures were performed laparoscopically. Following introduction of the da Vinci Xi® platform, the choice of LSC versus RAL depended on instrument availability and surgeons’ preference, with no specific selection criteria. All patients were preoperatively informed about the surgical technique, and the study was approved by the local ethics committee (Ethikkommission Nordwest- und Zentralschweiz (EKNZ), Project-ID 2020-01285).

### Data collection

Prospectively collected data were obtained from written hospital records, electronic databases as well as radiology reports. Demographic data, including age, sex and BMI, ASA scores, type of achalasia (I–III) based on high-resolution manometry, as well as outcome parameters were extracted. The latter included intraoperative complications, postoperative complications (30-day-morbidity), evaluated according to Clavien-Dindo classification [18], operation time, postoperative length of hospital stay (LOS), and postoperative length of intensive care unit (ICU) stay (LOI).

All patients were postoperatively contacted by telephone and asked to complete the gastroesophageal-reflux disease health-related quality of life (GERD-Hr-QuoL) questionnaire [19].

### Surgical technique

#### Laparoscopic approach

Patients were positioned supine in a reverse Trendelenburg position. The camera port was placed in a semi-open fashion according to Hasson, superior of the umbilicus and to the left, and pneumoperitoneum was established with 14 mmHg. Three assistant ports were placed in the left and right upper quadrant (12 mm left upper quadrant, 5 mm left below costal margin, 5 mm right upper quadrant) and a specifically designed subxyphoidal liver retractor was inserted. After mobilization of the lower esophagus and the gastric fundus, a cardiomyotomy of at least 10 cm was performed under endoscopic control. After thorough endoscopic exclusion of damage to the esophageal mucosa a 180° partial anterior fundoplication was performed according to Dor.

#### Robotic-assisted approach

**Fig. 1:**
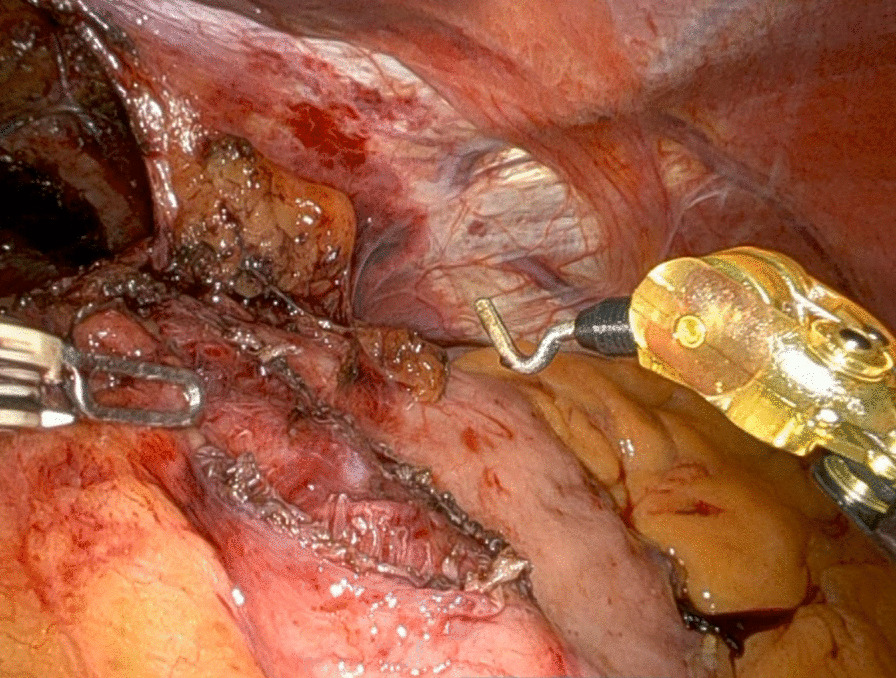
Heller myotomy

**Fig. 2:**
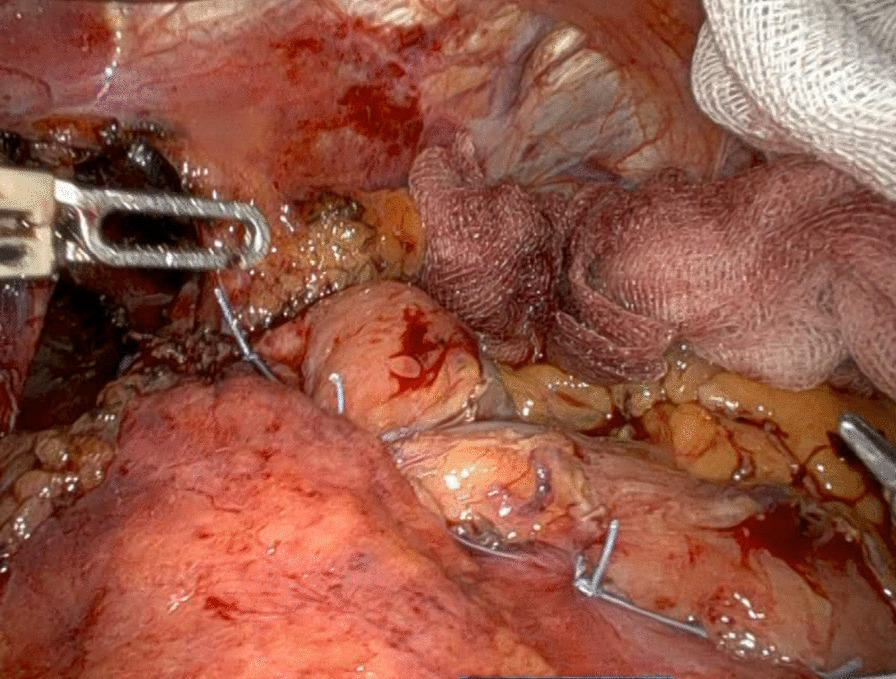
Dor fundoplicatio

The patient cart of da Vinci Xi® robotic platform (Intuitive Surgical, Sunnyvale, CA, USA) was placed at the patient’s head. Creation of pneumoperitoneum was performed using Veress needle and the first port was placed under visual control. Port placement was performed in a horizontal line above the umbilicus using four 8 mm da Vinci ports and 1 additional port. The surgical steps during the operation procedure were identical to LSC surgery (Figs. 1 and 2).

With the “Tile Pro Function” the laparoscopic as well as the endoscopic view are simultaneously visible for the surgeon, which seems a great advantage to exclude mucosal lesions in the robotic performed procedures.

In both patients’ groups the learning curve was included since data collection for laparoscopic procedures started in 2007 when laparoscopic procedures for achalasia were rather new and standardized techniques were evolving.

### Postoperative care

Following the implementation of robotic-assisted technology for HM, in general, almost every patient treated with RAL procedure was postoperatively precautionarily transferred to the intensive care unit (ICU) also including intermediate care unit and recovery room. An upper GI series with gastrographin was routinely performed in all patients usually at postoperative day one. Irrespective of surgical technique, all patients underwent a fasting period of variable duration prior to the initiation of enteral nutrition. A subsequent transition to a normal diet was performed starting with liquids depending on tolerance of food intake.

### Statistical analysis

Descriptive statistics were presented as absolute numbers and percentages for categorical variables, while continuous variables were presented as median and interquartile range (IQR). The comparison of categorical variables was performed with the chi-squared or Fisher exact test, while continuous variables were compared with the Mann–Whitney test. A p-value < 0.05 was considered statistically significant. Statistical analysis was performed on MedCalc® Statistical Software version 20.014 (MedCalc Software Ltd, Ostend, Belgium; https://www.medcalc.org; 2021).

## Results

### Patients’ characteristics

Demographics and clinical characteristics of the 32 patients treated with LSC and the 11 operated with RAL are reported in Table 1. Gender, median age and BMI were similar in the two groups. However, a trend (p = 0.052) towards a higher number of patients with ASA III score in the RAL-operated group was observed.

**Table 1:** Patient demographics and clinical characteristics

Groups characteristics	RAL N = 11	LSC N = 32	P
Median age, years (IQR)	60.5 (48.3–68.9)	54.9 (47.1–66.3)	0.351
Female gender, n (%)	6 (54.5)	9 (28.1)	0.117
BMI, kg/m^2^ (IQR)	23.1 (21.0–25.5)	23.7 (21.0–27.1)	0.802
ASA score			
• I, n (%)	3 (27.3)	9 (28.1)	0.052
• II, n (%)	3 (27.3)	19 (59.4)	
• III, n (%)	5 (45.5)	4 (12.5)	
Smoking			
• Never, n (%)	9 (81.8)	21 (65.6)	0.574
• Former, n (%)	1 (9.1)	7 (21.9)	
• Active, n (%)	1 (9.1)	4 (12.5)	
Achalasia type			
• I, n (%)	2 (18.2)	10 (34.5)	0.180
• II, n (%)	9 (81.8)	15 (51.7)	
• III, n (%)	0	4 (13.8)	
Preoperative symptoms			
• Dysphagia, n (%)	11 (100.0)	31 (96.9)	0.898
• Weight loss, n (%)	2 (18.2)	10 (34.5)	
• Pneumonia, n (%)	1 (9.1)	2 (6.2)	
Endoscopic dilation before surgery, n (%)	1 (9.1)	6 (18.8)	0.459

Values are presented as median with interquartile range (IQR) or absolute number with percentage in parentheses

*ASA* American society of anesthesiology, BMI: body mass index

Number of smokers (p = 0.574) and types of achalasia (p = 0.180) were similarly distributed in both groups. In addition, preoperative symptoms, including dysphagia, weight loss and pneumonia, were also similarly detectable. Furthermore, six patients of the LSC group and one of the RAL group (p = 0.459) had undergone endoscopic dilation prior to surgery.

### Surgical outcomes

No intraoperative complications occurred during LSC or RAL, and, in particular, no esophageal injuries occurred. Furthermore, no conversion to open surgery or conversion from RAL to LSC was necessary. However, operation time was significantly shorter in LSC than in RAL surgery (p = 0.047, Table 2).

**Table 2:** Peri- and postoperative outcomes

Groups characteristics	RAL N = 11	LSC N = 32	p
Operation time, minutes, median (IQR)	150 (150–187)	140 (136–150)	0.047*
Fasting, days, median (IQR)	5.0 (4.2–6.0)	5.0 (5.0–6.0)	0.576
Hospital stay, days, median (IQR)	11 (10–14)	11 (10–13)	0.712
ICU admission, n (%)	8 (72.7)	3 (9.4)	< 0.001***
Postoperative complications, Clavien-Dindo grade			
• 0, n (%)	4 (36.4)	22 (68.7)	0.045*
• I, n (%)	2 (18.2)	0	
• II, n (%)	4 (36.4)	9 (28.1)	
• III, n (%)	1 (9.1)	1 (3.1)	
Achalasia medications, n (%)	1 (9.1)	3 (9.4)	1.000

* *p* < 0.05, *** *p* < 0.001

Values are presented as median with interquartile range (IQR) or absolute number with percentage in parentheses

Postoperative complication rate was significantly (p = 0.045) lower in the LSC than in the RAL-operated group. However, in either case, most complications were of Clavien-Dindo grade I or II, and included infections (e.g. pneumonia and urinary-tract infections) which were treated with antibiotics, constipation and subcutaneous emphysema. Grade III complications only occurred in one patient of the LSC group and one of the RAL group (Table 2). Both consisted of pleural effusions requiring surgical insertion of a chest tube. No reoperations were necessary in either group.

Postoperative ICU admission rate was lower in the LSC, as compared to the RAL group (p < 0.001, Table 2). However, median postoperative length of precautionary ICU stay in the latter group (see above) was of 1 day. Instead, overall hospital stay was similar (p = 0.712) in the two groups.

No difference in the length of the postoperative fasting period could be detected, (p = 0.576), with a median duration of 5 days in either group. Moreover, similar numbers of patients needed achalasia medications following surgery (p = 1).

### Postoperative follow-up and quality of life

Due to the earlier adoption of laparoscopic procedure, median follow up was significantly longer in the LSC than in the RAL group (89 months, IQR: 45–129, vs. 28 months, IQR: 14.5–49.5, p = 0.0014). A total of 34 out of 43 patients (79%) returned the GERD-HRQL questionnaire (RAL 9/11, 82% and LSC 25/32, 78%), allowing a detailed analysis of quality-of-life.

Overall, no statistically significant differences between the LSC and RAL group could be observed (Table 3). In particular, median score was 4 in both LSC and RAL group. One patient (4.2%) in the LSC group felt “not satisfied”, whereas all patients in the RAL group were “satisfied” or “neutral” with the operation outcome. One patient in each group suffered from severe reflux, resulting in higher questionnaire scores. Notably, over one third of patients from either group needed proton pump inhibitors postoperatively (p = 0.561).

**Table 3:** GERD-health related quality of life questionnaire (GERD-HRQL)

Groups characteristics	RAL N = 9	LSC N = 25	P
Total, points, median (IQR)	4 (2–9)	4 (2–10)	0.969
• Reflux, points, median (IQR)	0 (0–3)	0 (0–6)	0.561
• Medication, points, median (IQR)	0 (0–0)	0 (0–0)	0.284
• Dysphagia, points, median (IQR)	2 (2–3)	1 (0–2)	0.130
• Regurgitation, points, median (IQR)	0 (0–5)	0 (0–3)	0.672
Satisfaction			
• Satisfied, n (%)	6 (66.7)	21 (87.5)	0.181
• Neutral, n (%)	3 (33.3)	2 (8.3)	
• Not satisfied, n (%)	0	1 (4.2)	
Need of proton pump inhibitors, n (%)	4 (44.4)	8 (33.3)	0.561

Values are presented as median with interquartile range (IQR) or absolute number with percentage in parentheses

## Discussion

Achalasia is a rare disease and a variety of pharmacological and surgical therapies of debated effectiveness have been proposed. HM currently represents the most effective achalasia treatment. However, different procedures have frequently been adopted even in a single center. While LSC and RAL have repeatedly been shown to be highly effective, comparative evaluations are difficult. Particularly due to very heterogeneous groups of patients are involved. Features impacting on clinical outcome, such as patient BMI or ASA scores, and type of fundoplication are frequently unreported [14, 20]. Most surprisingly, long term quality of life, a key outcome parameter, is rarely evaluated. To address these issues, we comparatively analyzed intra- and postoperative complication rates, and patients’ long-term (> 2 years) quality of life and satisfaction in two highly homogeneous groups of patients treated with standardized LSC or RAL HM in a single high-volume visceral surgery Swiss center.

Demographics of patients from the two groups were similar. However, a trend towards a higher number of patients with a higher ASA score in the RAL group was evident, suggesting a propensity of surgeons to operate more difficult cases with robotic technology.

In our series, operation time was marginally shorter in LSC, as compared to RAL HM (140 vs. 150 min, p = 0.047), possibly due to an evolving learning curve in RAL. These data closely match earlier reports from previous studies and meta-analyses [14, 17, 21, 22], documenting minor, if at all significant, differences.

In no case a conversion from RAL to LSC or to open surgery was necessary, consistent with literature data [14, 22]. Indeed, conversion has been shown to be more frequently required in re-operations for recurrent achalasia [23].

The rate of complications requiring an intervention was not significantly different in the two groups. In particular, one Clavien-Dindo grade III pleural effusions requiring surgical insertion of a chest tube was observed in each. These results compare favorably with data from Kim et al. [21] who observed a total of 5 esophageal perforations, 4 in the laparoscopic and 1 in the robotic-assisted group, with no statistically significant difference between the groups in 72 patients undergoing HM between 2006 and 2015 (LSC n = 35 and RAL n = 37). Although failing to achieve statistical significance, the lower number of intraoperative perforations in the RAL group was attributed to the technical advantages of robotic surgery, e.g. better vision and ameliorated motor control. In a more recent study [24], Ali et al. compared three groups of patients undergoing robotic, endoscopic and laparoscopic myotomy. In this series the rate of full-thickness injuries was also significantly higher in the laparoscopic group compared to the robotic-assisted or endoscopic group. A recent meta-analysis [15] corroborates these reports. In keeping with a large majority of previous reports [12, 13], we did not observe any mortality among our patients. Although the clinical relevance of intraoperatively recognized mucosal perforations during HM is debatable, unrecognized perforations may lead to reoperations with high morbidity and should be avoided [25].

Hospital stay of patients treated with LSC or RAL in our center was similar (p = 0.712), with a median of 11 days in either group. The rather long period without enteral nutrition (5 days) might explain, at least in part, this relatively prolonged stay. In this regard there is a wide heterogeneity in literature, probably also due to different health care systems [14, 15]. On the other hand, for safety reasons, after RAL HM, in general almost every patient was postoperatively transferred to the ICU, including intermediate care unit and recovery room, yet, on average, for 1 day only. This reflected a precautionary approach following the application of a new procedure rather than an actual clinical need.

The long follow-up period of our study has to be underlined because long-term data following HM is scarce and former studies suggest that initial favorable outcomes might worsen over time [26–28]. Median follow-up in our cohort was 89 months for the laparoscopic operated patients and 28 months for the robotically assisted operated patients (LSC 89 months IQR 45–129, RAL 28 months IQR 14.5–49.5). Patients’ long-term follow-up and postoperative symptoms were evaluated with a telephone interview using the GERD-Health Related Quality of Life Questionnaire (GERD-HRQL) [19], specifically developed to assess symptomatic outcomes for the typical symptoms of gastro-esophageal reflux disease. This questionnaire is recommended by the European Association of Endoscopic Surgery and is one of the most frequently used surveys for the documentation of symptom severity [29]. It consists of a total of 16 questions focusing on “heartburn”, “dysphagia”, “medication”, “regurgitation” and includes an important last question “How satisfied are you with your present situation”. By answering, a maximum of 75 points can be achieved and low scores indicate a better quality of life. In both LSC and RAL groups under investigation, the median number of points in the GERD-HRQL was 4 (LSC IQR: 2–10; RAL: IQR 2–9, p = 0.969), consistent with a low frequency of reflux and regurgitation or dysphagia. Most importantly, almost all patients were “satisfied” or “neutral”, with no difference between the LSC and RAL group. In this respect our results closely match those from previous studies with shorter follow-up times [29].

Limitations of our study need to be acknowledged. In particular, its retrospective and non-randomized nature might have affected data quality. In the RAL group, more ASA 3 patients were present, which can be attributed to a selection bias or a to trend of operating more high-risk patients in recent years. Moreover, there might have been a selection bias between the RAL and the LSC group, because the robotic platform was not always available during the whole study period. Finally, the number of patients is relatively modest.

However, achalasia is a rare disorder and even in centers with an active esophageal surgery program [30] the number of patients is small. Most importantly, the cohorts of patients analyzed are highly homogeneous, and they were operated in a single center by surgeons highly experienced in laparoscopic and robotic-assisted procedures, with a minor learning-curve effect. Therefore, our results reliably document a successful transition towards a surgical technology providing distinct advantages to the operator, while warranting clinical results and quality of life comparable to those obtained by using standard LSC procedures.

## Conclusions

Robotic-assisted laparoscopic HM for achalasia is safe and feasible. Intra- and postoperative outcome parameters and quality of life are comparable to those obtained following reference laparoscopic procedure. Many surgeons with high experience in both techniques now appear to prefer RAL over LSC for its higher precision, better and clearer vision, and, last but not least, improved ergonomic conditions for the operator. Therefore, while prospective, randomized, controlled multicenter trials are urgently required to provide high level evidence for the treatment of this rare condition and mitigate confounders, either approach can presently be recommended, depending on surgeons’ preference and experience.

## Data Availability

All data used and analyzed during the current study are available from the corresponding author on reasonable request.

## Abbreviations

LSC: Laparoscopic
HM: Heller myotomy
RAL: Robotic-assisted laparoscopic
GERD-Hr-QuoL: Gastroesophageal-reflux disease health-related quality of life
POEM: Per-oral endoscopic myotomy
BMI: Body mass index
ASA: American Society of Anesthesiologists
EKNZ: Ethikkommission Nordwest- und Zentralschweiz
LOS: Length of hospital stay
ICU: Intensive care unit
LOI: Length of intensive care unit stay
IQR: Interquartile range
